# The ON:OFF switch, σ1R-HINT1 protein, controls GPCR-NMDA receptor cross-regulation: Implications in neurological disorders

**DOI:** 10.18632/oncotarget.6064

**Published:** 2015-10-10

**Authors:** María Rodríguez-Muñoz, Elsa Cortés-Montero, Andrea Pozo-Rodrigálvarez, Pilar Sánchez-Blázquez, Javier Garzón-Niño

**Affiliations:** ^1^ Department of Molecular, Cellular and Developmental Neurobiology, Laboratory of Neuropharmacology. Instituto Cajal, Consejo Superior de Investigaciones Científicas (CSIC). Madrid, Spain

**Keywords:** σ1R, HINT1 protein, cannabinoid CB1 receptor, neurological disorders, mu-opioid receptor, Pathology Section

## Abstract

In the brain, the histidine triad nucleotide-binding protein 1 (HINT1) and sigma 1 receptors (σ1Rs) coordinate the activity of certain G-protein coupled receptors (GPCRs) with that of glutamate *N*-methyl-D-aspartate receptors (NMDARs). To determine the role of HINT1-σ1R in the plasticity of GPCR-NMDAR interactions, substances acting at MOR, cannabinoid CB1 receptor, NMDAR and σ1R were injected into mice, and their effects were evaluated through *in vivo, ex vivo,* and *in vitro* assays. It was observed that HINT1 protein binds to GPCRs and NMDAR NR1 subunits in a calcium-independent manner, whereas σ1R binding to these proteins increases in the presence of calcium. In this scenario, σ1R agonists keep HINT1 at the GPCR and stimulate GPCR-NMDAR interaction, whereas σ1R antagonists transfer HINT1 to NR1 subunits and disengage both receptors. This regulation is lost in σ1R^−/−^ mice, where HINT1 proteins mostly associate with NMDARs, and GPCRs are physically and functionally disconnected from NMDARs. In HINT1^−/−^ mice, ischemia produces low NMDAR-mediated brain damage, suggesting that several different GPCRs enhance glutamate excitotoxicity via HINT1-σ1R. Thus, several GPCRs associate with NMDARs by a dynamic process under the physiological control of HINT1 proteins and σ1Rs. The NMDAR-HINT1-σ1R complex deserves attention because it offers new therapeutic opportunities.

## INTRODUCTION

The prolific investigation of psychosis/schizophrenia and depression suggests that both G-protein coupled receptors (GPCRs) and glutamate *N*-methyl-D-aspartate receptors (NMDARs) participate in the pathophysiology of these mental illnesses; nevertheless, the hierarchy of these changes is still a matter of debate. Initially, depression was associated with alterations mostly in serotonergic and noradrenergic receptors [1, 2], and psychosis/schizophrenia was associated with dopamine and GABA receptors [3, 4]. Lately, the glutamatergic system has been considered as a determinant for the onset and consolidation of these dysfunctions, mostly because NMDAR activity increases in depressive subjects and decreases in patients suffering schizophrenia [1, 5].

The NMDAR is essential for the long-term potentiation and long-term depression of synapses that determine the weight that the incoming signals receive during these periods, GPCR signaling included. Therefore, NMDARs, by influencing the cellular impact of signals that are originated at GPCRs, would play an essential role in neuronal plasticity, development, differentiation, learning, and memory consolidation. Accordingly, NMDARs functionally recruit the negative control of certain GPCRs, such as the cannabinoid CB1, to prevent the risk of excitotoxicity [6]. In this context, the cellular impact of endocannabinoids on this glutamate ionotropic receptor is also under regulation; the calcium sensor σ1R [7] associates with the CB1-NMDAR complex and, when calcium levels are reduced, antagonists of σ1Rs disrupt the CB1-NMDAR association to prevent endocannabinoids from producing the hypofunction of NMDARs [8]. In vulnerable subjects with a defect in this molecular switch, cannabinoids could induce NMDAR hypofunction, bringing about symptoms of psychosis or even precipitating schizophrenia.

Thus, the relationship between GPCRs and NMDARs can work in both directions, and GPCR-triggered signaling cascades regulate NMDAR-mediated glutamate responses [9, 10]. GPCRs such as the mu-opioid receptor (MOR) [11], the dopamine D1 receptor [12], group I metabotropic glutamate receptors (mGluR1/5), group II mGluR2/3 [13, 14], and the serotonin 5HT2A/C receptor [15], recruit NMDAR activity through PLCβ- and PKC-mediated activation of the non-receptor tyrosine kinase Src. Other GPCRs reduce NMDAR function, *e.g.,* acetylcholine type 1 muscarinic receptor [16], serotonin 5HT1A receptor [17, 18], adrenergic α1 and α2 receptors [19], cannabinoid receptor 1 (CB1) [20], and group III mGluR7 [21].

The cytosolic C-termini of several GPCRs physically associate with NMDAR NR1 subunits, *i.e*., dopamine D1 receptors [22], group I metabotropic glutamate receptors (mGlu5a) [23], MOR [24], and CB1 [25]. The C-terminus of the NR1 subunit is composed of C0-C1-C2(C2´) regions; however, some NR1s lack the C1 segment [26]. Because the GPCR C-termini interact with NR1 subunits carrying the C1 region, NMDARs containing just NR1 C0-C2(C2′) regions would be excluded from such direct regulation by GPCRs. The tandem integrated by the histidine triad nucleotide-binding protein 1 (HINT1) and σ1R is essential to connect GPCRs such as MOR and CB1 receptors with NMDAR function (Table I). In mice lacking either of these proteins, morphine does not recruit NMDAR function, and the direct activation of NMDARs does not reduce morphine analgesia [27, 28]. Accordingly, in these mice, cannabinoids do not exert the expected negative control on NMDAR-mediated calcium influx or zinc metabolism [25].

**Table 1:** Relevance of HINT1 proteins and σ1Rs in GPCR-NMDAR cross-regulation

**HINT1^−/−^ mice**	**References**
Impaired association of MOR with the NMDAR NR1 subunit	[49]
Enhanced morphine antinociception	[27, 42]
Enhanced and NMDAR-independent antinociceptive tolerance	[27, 49]
Heterologous tolerance	[49]
NMDA does not antagonize morphine antinociception	[49]
Cannabinoids do not reduce NMDAR activity	[25, 17]
HINT1 restores CB1 protection against excitotoxicity	[17]
Impaired association of CB1 with NMDAR NR1 subunit	[25]
Anti-depressant and anxiolytic-like behaviors	[39]
Dysregulated postsynaptic dopaminergic transmission	[38]
**The HINT1 protein and neurological disorders**	
*HINT1* gene is a candidate for schizophrenia	[29, 30, 31]
Association of the HINT1 gene with nicotine dependence	[36, 37]
**σ1R^−/−^ mice**	**References**
Impaired association of MOR with the NMDAR NR1 subunit	[28]
Enhanced morphine antinociception	[28, 71, 72]
Nearly absent allodynia	[56, 90]
Enhanced and NMDAR-independent antinociceptive tolerance	[28]
Heterologous tolerance	
NMDA does not antagonize morphine antinociception	
The σ1R restores MOR-NMDAR cross-regulation	
Cannabinoids do not reduce NMDAR activity	[8]
NMDAR activity does not recruit CB1 control	
Impaired association of CB1 with the NMDAR NR1 subunit	
**The σ1R and neurological disorders**	
The ***σ1R*** gene is a candidate for schizophrenia	[32, 33, 34, 35]
σ1R ligands are antidepressants and anxiolytics	[40, 41]

In humans, the *HINT1* and *σ1R* genes have been implicated in schizophrenia [29, 30, 31, 32, 33, 34, 35], and mice lacking the HINT1 protein show an altered dopamine transmission that could mediate their tendency to drug abuse [36, 37]. These HINT1^−/−^ mice show antidepressant and anxiolytic-like behaviors [38, 39] and, importantly, σ1R ligands promote antidepressant and anxiolytic-like behaviors in wild-type mice [40, 41]. All of these observations led us to propose that in neural cells, HINT1 and σ1R work together to maintain the cross-regulation between a series of GPCRs and NMDARs that is necessary for the successful integration of their concurrent signals into cell metabolism.

The precise characterization of such a molecular mechanism could provide valuable information on how certain GPCRs and NMDARs coordinate their activities and would help detect whether anomalies of this regulatory process contribute to neurological disorders, providing new therapeutic targets. With this aim, we investigated the role of the σ1R putative endogenous ligands, neurosteroids, in the association of the HINT1 protein with MOR/CB1 receptors and NR1 C1 subunits. We sought to determine whether this tandem of proteins works as an on-off switch under the regulation of σ1Rs and calcium levels, which mostly reflect the activity of NMDARs in this environment.

## RESULTS

### The association of HINT1 proteins and σ1Rs with GPCRs

**Figure 1:**
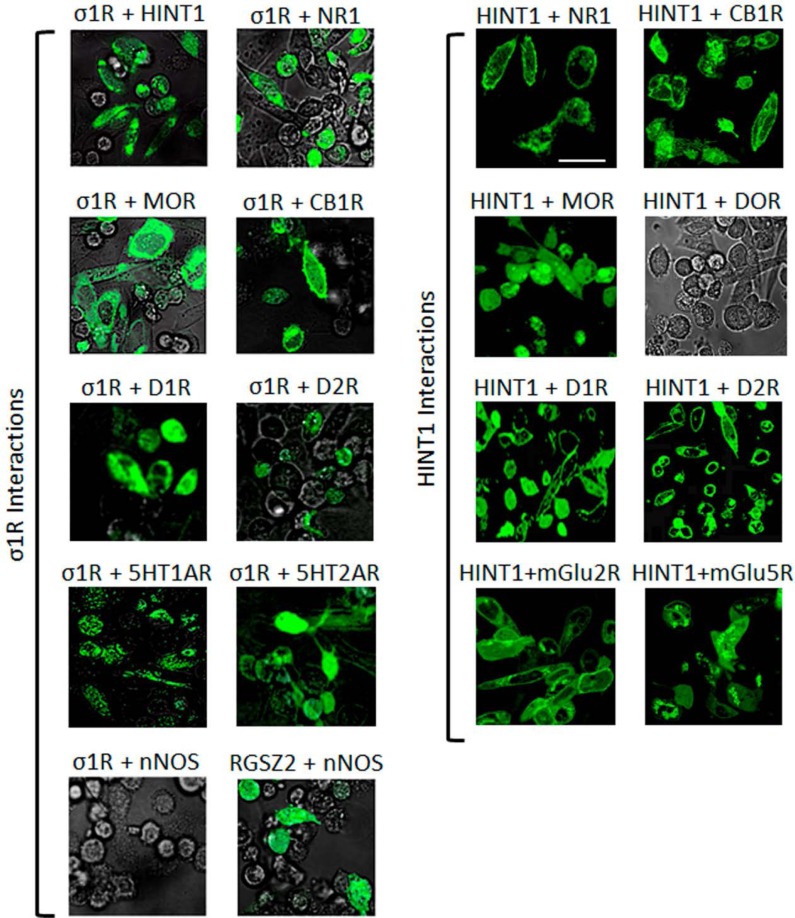
Interactions of σ1Rs and HINT1 proteins with different GPCRs and NMDAR NR1 C0-C1-C2 subunits Visualization of the interactions by BiFC. CHO cells were transiently co-transfected with cDNAs encoding the pair of full-length proteins of interest in the VN173 and VC155 plasmids (0.3 μg), and confocal fluorescent signals were obtained 24 h later when VN173 and VC155 had associated. Scale bar: 10 μm. The σ1R associates with diverse GPCRs, HINT1 and NMDAR NR1 subunits that contain the C1 cytosolic segment. The nNOS is brought to the MOR environment through its binding to RGSZ2 [46]. Thus RGSZ2 and nNOS show interaction, whereas σ1R and nNOS do not (negative control). HINT1 interacts with several different GPCRs; however, its interaction with the delta opioid receptor (DOR) is very weak.

At the plasma membrane, the HINT1 protein and the σ1R associate with the NMDAR NR1 subunit [28] and the MOR [42, 43], a finding that has been extended to other GPCRs [44, 45]. Using bimolecular fluorescence complementation (BiFC) in living cells, we demonstrated that as well as the MOR, HINT1 and σ1R can associate with the cannabinoid CB1, dopamine D1 and D2, serotonin 1A and 2A, and metabotropic glutamate 2 and 5 receptors. Notwithstanding, in living cells, the delta-opioid receptor (DOR) did not interact with HINT1 (Figure 1). Indeed, the HINT1 protein in mouse brain synaptosomes co-precipitates with MORs and CB1 receptors but not with DORs [45].

The HINT1 protein and the regulator of G protein signaling of the Rz subfamily, RGSZ2 (also named RGS17), are endogenous to CHO cells. RGSZ2 couples to neural nitric oxide synthase (nNOS) and regulates its activity (positive BiFC interaction). While RGSZ2 and the σ1R bind to the HINT1 protein [46], a nNOS interaction with σ1Rs was not evident in the BiFC assay. Thus, our experimental conditions did not favor the indirect interaction of the target proteins within protein complexes (see Methods).

**Figure 2:**
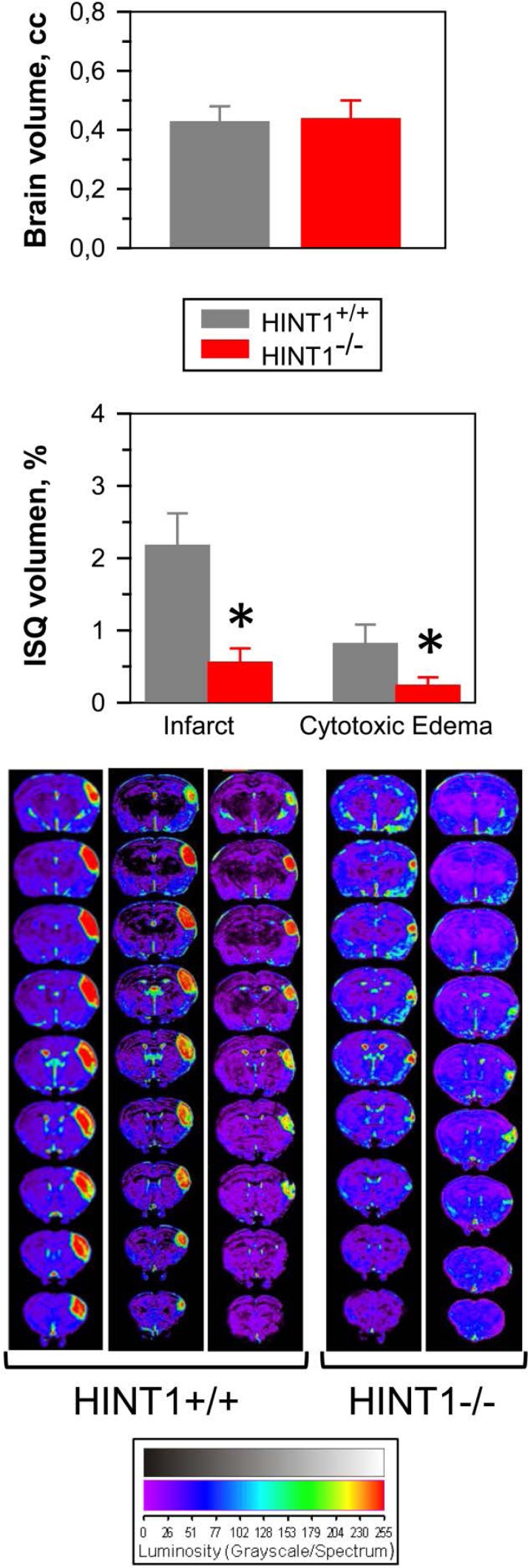
The absence of HINT1 diminishes ischemic brain damage Representative brain section images from wild-type and HINT1^−/−^ mice obtained 48 h after MCAO. Knockout animals have significantly smaller infarct areas. We used the dorsal third ventricle as an internal anatomical marker from wild-type and HINT1^−/−^ mice to align, register, and compare the collection of images from each mouse. The infarct volume was calculated as the percentage of the hemisphere that is infarcted. Groups were of 8-10 mice, and the data are represented as the mean ± S.E.M. The average volumes of whole brain are similar in both groups of mice. The bar graphs shown below quantitatively compare the edema and infarct volume (± S.E.M.) from HINT1+/+ (grey bars) and HINT1−/− (red bars). *Significantly different, paired t test, degrees of freedom (DF) = 16, *p* < 0.05.

The influence of GPCRs on the activity of NMDARs was also studied in a model of focal ischemia in mice, permanent occlusion of the middle cerebral artery. In HINT1^−/−^ mice, brain damage was practically restricted to the region directly irrigated by the occluded artery (see the anatomical location of the damage in consecutive MRI slices from the caudal to rostral planes in Figure 2). The average volumes of the whole brain was similar in both groups of mice, yet compared to the wild-type mice, the infarct and surrounding cytotoxic edema was difficult to detect in HINT1^−/−^ mice. This brain damage is mostly caused by secondary overactivation of NMDARs [47, 48] and thus, the absence of HINT1 could prevent focal-activated GPCRs from enhancing NMDAR activity in the surrounding area, diminishing the overall ischemic damage. In fact, there is a functional and physical disconnection between GPCRs (such as the MOR, CB1, serotonin 5HT2A or dopamine D2 receptors) and the NMDAR in HINT1^−/−^ mice [17].

### The complex MOR-HINT1-σ1R-NMDAR NR1 subunit

**Figure 3:**
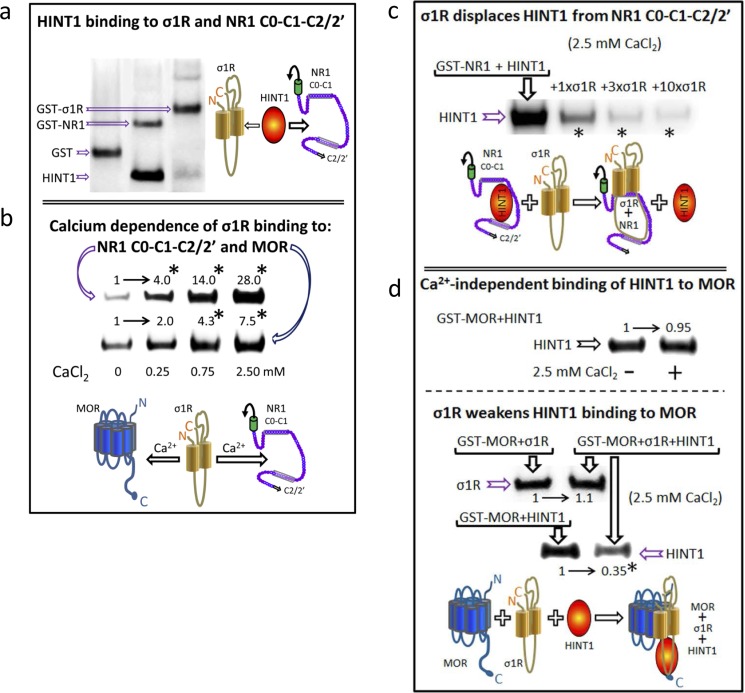
HINT1 and σ1R binding to the MOR and NMDAR NR1 subunit C-terminal sequence C0-C1-C2 **a.** *In vitro HINT1 binding to σ1R and NR1 subunits*. Because HINT1 forms homodimers, the protomer was used at 200 nM, whereas the GST-σ1R and GST-NR1 C0-C1-C2 peptides were used at 100 nM (GST alone did not bind to the HINT1 protein: lane 1, negative control). Glutathione Sepharose 4B captured the GST fusion protein, and the pellets were then washed, solubilized in 2x Laemmli buffer and resolved by SDS-PAGE. The presence of HINT1 and GST was analyzed sequentially in Western blots (WB). **b.** *Effect of calcium on the association of* σ1R *with NR1 subunits and MORs*. The recombinant proteins were used at 100 nM. The assay was performed in the presence of increasing amounts of calcium chloride (0, 0.25, 0.75, or 2.5 mM). Bait proteins (GST-NR1 C0-C1-C2 and GST-MOR) were immobilized by covalent attachment to NHS-activated Sepharose. Prey protein (σ1R) alone did not bind to either the NHS-Sepharose or the recombinant GST (negative controls). The pellets obtained were processed as described. *Significantly different from the immuno-signals of the 0 mM CaCl_2_ group assigned an arbitrary value of 1; ANOVA, total DF = 15, followed by Dunnett multiple comparisons *vs* control group, *p* < 0.05. **c.** *The σ1R displaces HINT1 from its binding to NR1 subunits.* Competition experiments were conducted to study the possible interference between HINT1 and *σ*1R in their binding to the NR1 C-terminal sequence C0-C1-C2. The HINT1 protein (200 nM) was incubated with agarose-NR1 C0-C1-C2 for 40 min at RT in 150 μL of Tris-HCl 50 mM, pH 7.5, 2.5 mM CaCl_2_, and 0.2% CHAPS (TCaCh). After removal of free HINT1, increasing amounts of σ1R (100 nM, 300 nM, or 1 μM) were added. *Significantly different, ANOVA (total DF = 11), Dunnett multiple comparisons *vs* control group (no σ1R), *p* < 0.05. *(d) The σ1R reduces HINT1 binding to the MOR.* Upper panel, HINT1 binds to the MOR in a calcium-independent manner: t test (DF = 4) *p* > 0.05. Agarose-MOR was incubated with HINT1 (200 n*M*) in the presence or absence of 2.5 m*M* CaCl_2_ (30 min, RT). Lower panel, whilst HINT1 does not alter σ1R binding to the MOR [paired t test (DF = 4) *p* > 0.05], the σ1R reduces the HINT1-MOR association: *Significantly different, paired t test (DF = 4), *p* < 0.05. Agarose-MOR carrying associated HINT1 was incubated with TCaCh buffer in the absence or presence of 300 nM σ1R. Agarose was recovered and washed before the analysis of the MOR-bound HINT1/σ1R through SDS-PAGE and WB.

The GPCRs actually reported to physically interact with NMDARs bind to NR1 subunits that contain the C1 segment in their cytosolic C-terminal sequence. The HINT1 protein and the σ1R also bind to this region of the NR1 subunit [28, 25], again suggesting their participation in the cross-regulation that GPCRs establish with NMDARs. BiFC assays revealed that HINT1 establishes direct interactions with σ1Rs and NR1 C1 subunits (Figure 1). Notwithstanding, the HINT1 protein binds to NR1 subunits with greater affinity than to σ1Rs (Figure 3a). In the endoplasmic reticulum, the binding of σ1R to the immunoglobulin protein BiP requires calcium [7]. Accordingly, at the plasma membrane level, the interaction of σ1Rs with MORs and NMDARs was also promoted by calcium. In the absence of calcium, σ1R still displayed binding to MORs; however, it bound poorly to NR1 subunits. Increases in calcium levels up to 2.5 mM progressively enhanced the association of σ1Rs with both NR1 subunits and MORs (Figure 3b). Calcium influences the conformation of the σ1R C-terminus to form, together with the two transmembrane domains, the neurosteroid binding-site. This calcium-sensitive region of the σ1R disrupts intramolecular hydrophobic interactions in the NR1 C-terminal cytosolic sequence [28]. Thus, our data suggest that σ1Rs and MORs (GPCRs) interact through their respective hydrophobic transmembrane regions.

In their association with NR1 subunits, the σ1R prevails over the HINT1 protein (Figure 3c); σ1R just weakens the HINT1 interaction with MORs. However, HINT1 apparently does not affect the association of MOR with the σ1R (Figure 3d). Therefore, the *in vitro* studies provided valuable information on how these signaling proteins could regulate the functional relationship between GPCRs (MOR) and NMDARs *in vivo*.

### The σ1R controls HINT1 transfer from the MOR towards the NMDAR NR1 subunit

**Figure 4:**
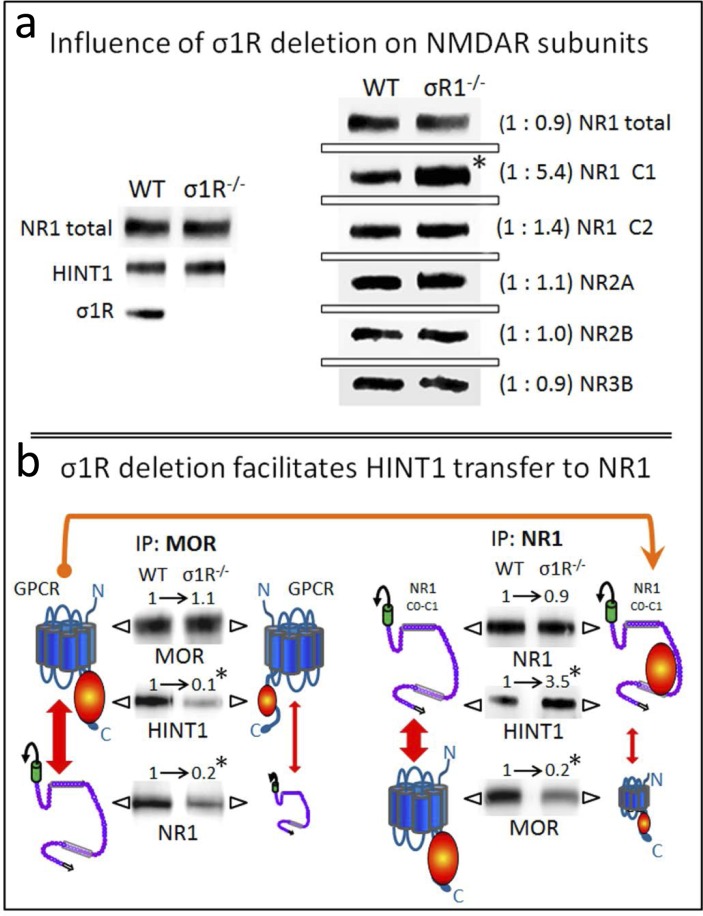
Influence of targeted deletion of the σ1R gene on HINT1-dependent MOR binding to NR1 subunits **a.** *The influence of* σ*1R deletion on NMDAR subunits.* The presence of NR1 subunits, HINT1 and σ1R in the synaptosomes of cerebral cortex obtained from wild-type and *σ*1R^−/−^ mice. The mice were euthanized, and the synaptosomes were obtained from the cerebral cortex and processed to obtain the membrane (P2 fraction: see the Methods section). Equal loading was verified and, where necessary, the data from direct detection assays were adjusted using the actin signals. The assay was repeated three times; mean ratios are shown in brackets. An arbitrary value of 1 was assigned to the wild-type group (WT). *Significantly different, paired t test (DF = 2), *p* < 0.05. **b.** *Deletion of σ1R favors HINT1 transfer from MORs towards NMDAR NR1 subunits.* The MORs or NMDAR NR1 subunits were subjected to immunoprecipitation (IP), and the co-immunoprecipitated proteins were assessed in WB. The presence of MORs and NR1 subunits was related to the IgG signals. MOR and total NR1 subunits did not differ between WT and *σ*1R^−/−^ mice, and these signals were used as the loading controls for the co-immunoprecipitated proteins. The width of the vertical red arrows indicates the association of MORs with NMDAR NR1 subunits. The experiments were repeated three times using membranes from different groups of mice. Antibody binding was visualized through chemiluminescence (ChemiImager IS-5500 system) and measured (Quantity One Software, Bio-Rad; average optical density of the pixels within the object area/mm^2^). For IP of the MOR or NR1: *significantly different from the immuno-signals of the WT group assigned an arbitrary value of 1; ANOVA (total DF = 17), all pairwise Holm-Sidak multiple comparison test, *p* < 0.05.

Our observations suggest that HINT1 binding to MORs and NMDARs is under regulation by σ1Rs. Therefore, we studied the influence of the targeted deletion of the *σ1R* gene in the association of HINT1 with these receptors. The σ1R^−/−^ mice exhibited NR1, NR2, NR3 and HINT1 levels comparable to those of the wild-type mice. However, in these knockout mice, the NR1 variant that contains the C1 segment was increased 5-fold compared to the NR1 variant lacking this segment (Figure 4a). The C1 region of NR1 subunits couples GPCRs and binds to HINT1/σ1R, thus this change might be compensatory, seeking to increase the probability of establishing such associations.

In fact, the *ex vivo* analysis of the relationship between MOR and NR1 subunits revealed that the MOR-NR1 association diminishes in σ1R^−/−^ mice, as already observed for the CB1-NR1 association [8]. Notably, HINT1 shows little binding to MORs despite its increased presence at the NR1 subunits (Figure 4b). In wild-type mice, σ1Rs likely maintain HINT1 proteins at the MOR where they stimulate the association of the GPCR with NMDARs.

### HINT1 swapping between GPCRs and NR1 subunits is a physiological process regulated by ligands of the σ1R

**Figure 5:**
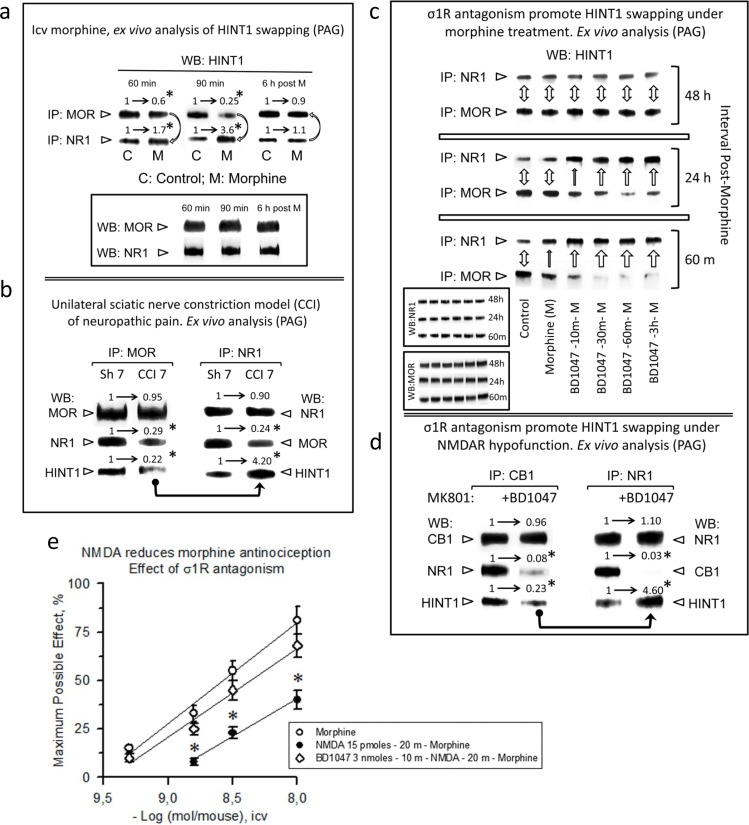
Physiological HINT1 transfer to NR1 subunits: role of σ1R antagonism **a.** *Morphine induces HINT1 swapping between MORs and NMDAR NR1 subunits*. CD1 mice received an icv injection of 10 nmol morphine and were euthanized at the post-opioid intervals indicated. The MORs or NR1 subunits were subjected to IP from PAG synaptosomes, and the co-precipitated HINT1 proteins were immunodetected by WB. The experiments were repeated three times; mean ratios are shown. For IP of the MOR or NR1: *significantly different from the immuno-signals of the control (C) group that received saline instead of morphine (M) and was assigned an arbitrary value of 1; ANOVA (total DF = 17), all pairwise Holm-Sidak multiple comparison test, *p* < 0.05. **b.** *Influence of neuropathic pain (CCI animal model) in HINT1 binding to MORs and NR1 subunits*. The mice were operated on day 0, and they were euthanized 7 days later. Following IP of MORs or NR1 subunits from PAG synaptosomes, the co-precipitated proteins were determined by WB. *Significantly different from the sham-operated control group (Sh7), *p* < 0.05. Details as in (a). **c.** *σ1R antagonism promotes HINT1 swapping with morphine treatment*. The mice received 3 nmol of the σ1R antagonist BD1047 and 10 nmol of morphine by the icv route, and different groups were euthanized at the post-opioid intervals indicated. The procedure was as described in (a). The bidirectional arrow indicates that the level of HINT1 association to MOR and NR1 subunits has returned to that observed in the control mice (no morphine). One top arrow indicates the transfer of HINT1 towards NR1 subunits, and its width is an arbitrary representation of the intensity of the process. Inset: WB of the IP MORs and NR1 subunits that served to determine the co-precipitated HINT1. **d.** *On NMDAR hypofunction, σ1R antagonism transfers HINT1 from CB1 receptors to NR1 subunits*. Mice received icv saline or 3 nmol BD1047 25 min before receiving 1 nmol MK801 (NMDAR antagonist), and the mice were euthanized 30 min later. Samples were processed as in b, except that CB1 was the immunoprecipitated GPCR, and the control group received MK801 but not the *σ*1R antagonist BD1047. For IP of CB1 or NR1: *significantly different from the MK801 group not receiving BD1047, *p* < 0.05. Details as in (a). **e.** *σ1R antagonism prevents NMDA from reducing morphine antinociception*. The morphine dose-effect curve for analgesia in mice was constructed. The icv injection of 15 pmol NMDA 20 min before morphine reduced the capacity of morphine to produce antinociception. The antagonist of σ1R, BD1047 (3 nmol), injected icv 10 min before NMDA (15 pmol), prevented the effect of the direct activator of NMDARs. *Significantly different from the control group that received morphine and saline instead of BD1047 and NMDA; ANOVA (total DF = 29), all pairwise Holm-Sidak multiple comparison test, *p* < 0.05.

The transfer of HINT1 from MORs to NMDARs results in the impairment of their cross-regulation, *i.e*., the activation of MORs does not recruit NMDAR activity, and that of the NMDAR does not impair MOR-mediated effects, such as antinociception [49]. To prevent an excess of NMDAR signaling that could promote excitotoxicity, the activity of this receptor is under exquisite control by endogenous systems, such as the endocannabinoid system [6]. To test whether the GPCR-mediated enhancement of NMDAR function is regulated by such a mechanism, we analyzed the possibility of HINT1 swapping for disrupting the GPCR-NMDAR relationship. A single icv dose of 10 nmol morphine produces an analgesic effect in mice that peaks after 30 min and is still present 60-90 min later. This dose of the opioid promoted some transfer of HINT1 proteins from the MORs to the NR1 subunits, which reverted after the analgesic effects disappeared (Figure 5a). We also analyzed this molecular mechanism in an animal model of neuropathic pain, the sciatic nerve chronic constriction injury (CCI), where an anomalous activation of NMDARs led to the windup sensitization of nociceptive pathways [49]. Seven days after surgery, the mice exhibited reduced MOR-NR1 association, and HINT1 was found mostly bound to NR1 subunits (Figure 5b).

A previous study suggested a regulatory role for σ1R antagonists but not for σ1R agonists on HINT1-mediated disruption of the MOR-NMDAR association [28]. To confirm and extend the hypothesis that the σ1R regulates the swapping of HINT1 between GPCRs and NMDARs, we studied the effect of another compound that increases morphine analgesia and has been classified as an antagonist of the σ1R. Thus, BD1047 administered before morphine greatly increased the transfer of HINT1 to the NR1 subunit. In these circumstances, the return of HINT1 to the MOR required approximately 48 h (Figure 5c). The concept that HINT1 transfer uncouples the function of NMDARs from that of GPCRs is also supported by σ1R antagonists disrupting cannabinoid CB1 negative regulation of NMDAR function. After the NMDAR antagonist MK801 promoted the hypofunction of this glutamate ionotropic receptor, σ1R antagonism disconnected the negative control that CB1 receptors exert on NMDAR function [8], and we have now demonstrated that this effect was achieved by promoting HINT1 swapping to NR1 subunits and disruption of the CB1-NR1 association (Figure 5d).

Consistent with the negative control that NMDAR activity exerts on MOR signaling, the icv administration of NMDA reduces the capacity of morphine to promote analgesia [24]. In this scenario, σ1R antagonism restored morphine antinociception by preventing the negative effect of activated NMDARs on MOR signaling (Figure 5e). Again, the appropriate regulation of σ1R resulted in the uncoupling of the NMDAR from the GPCR's activity.

### Calcium and neurosteroids regulate the association of σ1Rs with GPCRs and NMDARs

**Figure 6:**
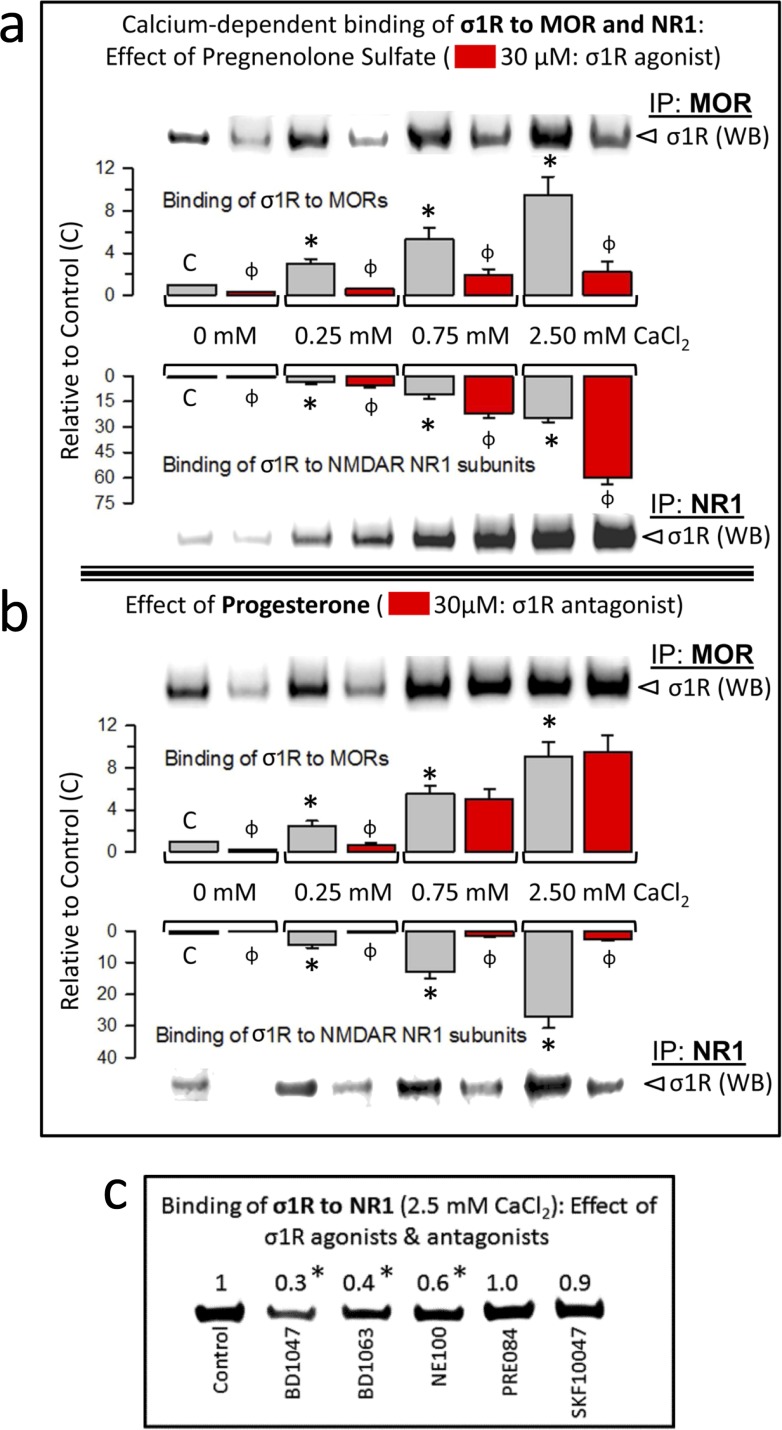
Calcium-dependent binding of σ1Rs to MORs and NR1 subunits: Influence of σ1R regulation **a.** *The σ1R agonist pregnenolone sulfate stabilizes the σ1R-NR1 interaction while diminishing σ1R binding to MORs*. The recombinant MOR, NR1 C0-C1-C2 and σ1R were used at 100 nM. The assay was performed in the presence of increasing amounts of calcium chloride (0, 0.25, 0.75, 2.5 mM). Bait proteins (GST-NR1 C0-C1-C2 and GST-MOR) were immobilized by covalent attachment to NHS-activated Sepharose. Prey proteins alone did not bind either to the NHS-Sepharose or to the recombinant GST (negative controls). The pellets obtained were processed as described to determine σ1Rs in Western blots (see the Methods section). The bars are the mean ± S.E.M of three independent assays. Effect of calcium. For each interaction of σ1R, MOR-σ1R and NR1-σ1R, the effects of increasing calcium availability are shown relative to the data obtained in the absence of calcium control group (C): arbitrary value of 1): *Significant differences, ANOVA (DF = 11), Dunnett multiple comparisons *vs* control group, *p* < 0.05. Effects of σ1R agonism (bars in red). These effects are indicated for each interaction of σ1R and calcium concentration studied: ^ɸ^ significant difference between the paired groups at each calcium concentration studied, with and without the sigma ligand; ANOVA (DF = 23) all pairwise Holm-Sidak multiple comparison test, *p* < 0.05. **b.** While diminishing σ1R binding to NR1 subunits, Progesterone, a σ1R antagonist, has a calcium-dependent effect at σ1R-MOR complexes. Details as in (a). **c.** Effect of exogenous (synthetic) ligands of σ1Rs on the NR1-σ1R complex: agonists, PRE084 and SKF10047; antagonists, BD1047, BD1063 and NE100. *Significantly different from the control group that received saline instead of the σ1R ligand; ANOVA (total DF = 11), Dunnett multiple comparisons *vs* control group, *p* < 0.05.

Neurosteroids are the putative endogenous ligands of the σ1R; among these, pregnenolone sulfate and progesterone behave as an agonist and an antagonist, respectively [see *e.g.*, 28]. We studied the effects of these steroids on the calcium-dependent association of σ1Rs with GPCRs (MOR) and NMDARs (NR1 C1 subunits). Pregnenolone sulfate reduced σ1R-MOR association over the range of calcium concentrations studied and increased the σ1R-NR1 interaction (Figure 6a). Additionally, progesterone at calcium concentrations up to 0.25 mM reduced σ1R-MOR association, an effect that disappeared in the presence of 0.75 mM and 2.5 mM calcium. The σ1R antagonist greatly reduced the σ1R-NR1 interaction independently of calcium levels (Figure 6b). The effect of neurosteroids on the association σ1R-NR1 was also produced by exogenous ligands of σ1R. Thus, the antagonists BD1047, BD1063 and NE100 reduced this interaction, and the agonists PRE084 and SKF10047 did not alter the interaction (Figure 6c).

## DISCUSSION

**Figure 7:**
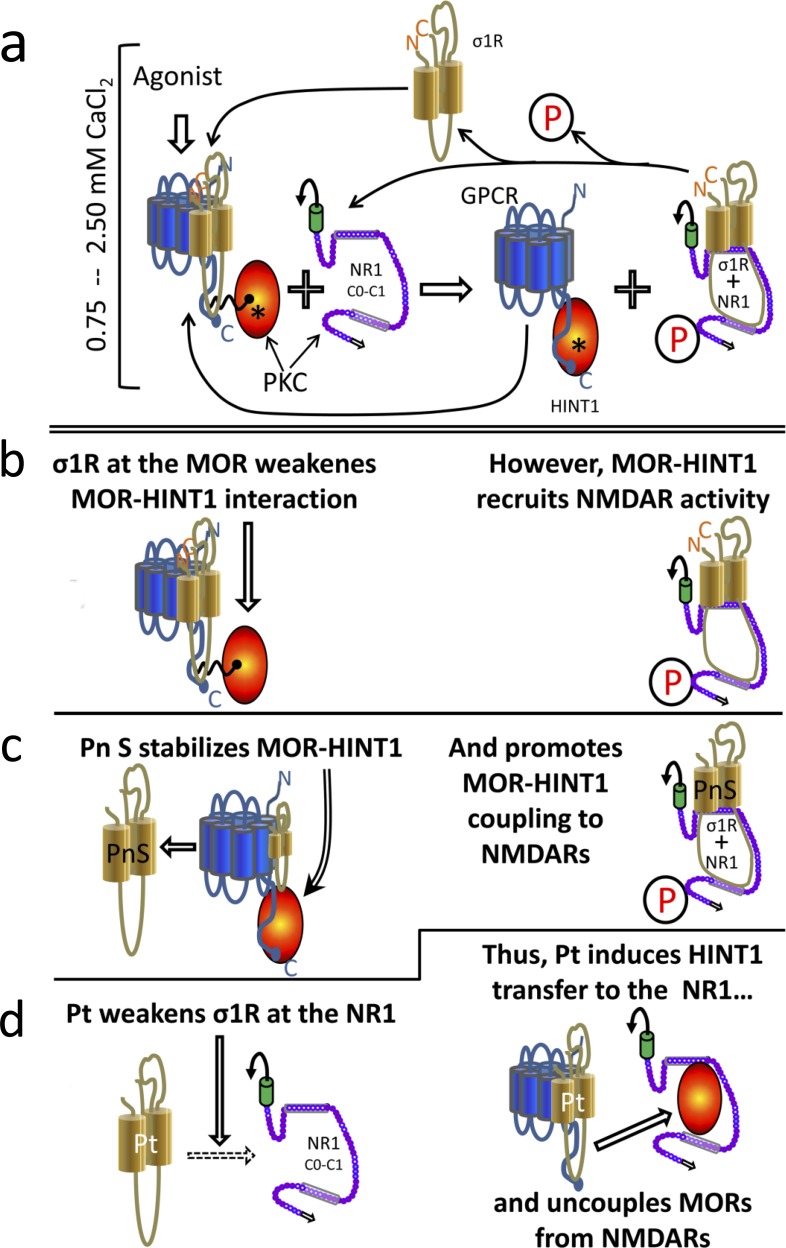
Diagram showing the σ1R- and calcium-dependent swapping of HINT1 proteins between GPCRs (MOR) and NMDAR NR1 subunits Physiological cellular levels of calcium are considered (between 0.75 mM and 2.5 mM). In the starting situation **a.** and **b.**, the MOR simultaneously binds to the σ1R and to the HINT1 protein. In this complex σ1R weakens the MOR-HINT1 interaction. The binding of RGS-Rz proteins, such as RGSZ1 and RGSZ2, to HINT1 prevent the association of the MOR-HINT1 complex with NMDAR NR1 subunits. The activation of MORs brings about the activation of PKC through PLCβ that increases calcium and diacylglycerol levels. Then, PKC releases RGS-Rz proteins and favors the binding of MOR-HINT1 to NMDAR NR1 C1 [see 46, 28]. PKC, acting on NR1 C1 serines 890 and 896, releases the NMDAR from the MOR-HINT1 inhibitory association, and PKC-recruited Src acts on tyrosine residues of the NMDAR NR2 subunits. The activated NMDAR greatly increases calcium levels in its environment, which promotes the transfer of the σ1R from the MOR towards the activated NMDAR NR1 subunit. The absence of the σ1R reinforces the MOR-HINT1 association, which is now ready to interact with other σ1Rs and to recruit the function of additional NMDARs. The activated NMDAR cannot bind to the MOR-HINT1 complex because of σ1R bound to the NR1 and the phosphorylation of the NR1 C1 segment. When MOR activity decreases, phosphatases reduce NMDAR-mediated calcium influx [89] and the calcium-dependent σ1R-NR1 association diminishes. Then, Ca^2+^-calmodulin gains access to NR1 subunits and sets NMDAR activity to a minimum. The removal of agonist-bound σ1Rs from the MOR environment stabilizes the MOR-HINT1 association and facilitates the recruitment of NMDAR activity **c.** Thus, σ1R agonists can increase the speed at which NMDAR activity is recruited by MORs. However, σ1R antagonists keep the σ1R bound to the MOR, weakening the MOR-HINT1 interaction while preventing σ1R binding to the NR1 subunit; both simultaneous actions make it possible for HINT1 to exit the MOR towards the NR1 subunit **d.** Thus, the same molecular machinery couples and uncouples the function of GPCRs such as MOR/CB1 to that of NMDARs. This outcome is achieved by the regulation of σ1Rs with agonists and antagonists. PKC (protein kinase C); P stands for phosphorylation of serine/threonine residues; Pn S (pregnenolone sulfate, σ1R agonist); Pt (progesterone, σ1R antagonist).

In the nervous tissue, NMDARs coordinate their function with that of GPCRs to regulate the efficacy of the synaptic function. Thus, there is an increasing interest on these interactions and whether the association of GPCRs with NMDARs described in different neural areas could be also implicated in these processes [10]. The present study shows that the physical interaction between NMDARs and GPCRs, such MOR and CB1, supports their cross-regulation *via* the molecular mechanism integrated by the HINT1 protein and the σ1R. These proteins work as a switch that responds to calcium levels and regulators of the σ1R. The agonists of the σ1R keep the HINT1 protein at the GPCRs and favor their interaction with NMDAR NR1 subunits -on situation. On the contrary, σ1R antagonists swap HINT1 from the GPCR to the NMDAR and disconnect both receptors -off situation (Figure 7). Thus, GPCR-NMDAR associations obey a dynamic process in which the HINT1-σ1R tandem is essential for GPCRs to connect with NMDAR function, and in the absence of either of these proteins, their relationship becomes disrupted.

The CB1 receptor is one of the most abundant GPCRs in the nervous tissue; although its localization is mostly presynaptic the CB1 receptor is also present in somata and dendrites [50, 51]. The presence of NMDARs in the presynapse [52] enable the physical association of CB1s with NR1 subunits at both sides of the synaptic cleft. GPCRs that activate PLCβ can recruit NMDAR function *via* PKC and Src (see the Introduction); thus, GPCRs *via* PLCβ/calcium and NMDARs *via* calcium influxes promote the release of endocannabinoids from the postsynapse to act on pre- and post-synaptic CB1 receptors [53]. This action regulates synaptic tone and connects the negative control of cannabinoids to NMDAR signaling [6].

It is certain that cannabinoids can reduce the impact of NMDARs by regulating signaling pathways that converge intracellularly with those triggered by the glutamate receptor. Nevertheless, in the absence of HINT1 or σ1Rs, NMDARs elude the control of cannabinoids, suggesting that the CB1-NMDAR association regulated by HINT1-σ1R is essential for cannabinoids to exert their negative control on NMDAR-mediated calcium influx, zinc metabolism and excitotoxicity. Indeed, the expression of these proteins in HINT1^−/−^ and σ1R^−/−^ deficient mice restores the cross-regulation between MOR/CB1 and NMDARs [28, 17]. Conceivably, a similar molecular mechanism might operate between other GPCRs and NMDARs. Indeed, the positive regulation of NMDAR activity by group I metabotropic glutamate receptors [13, 54] and certain serotonergic receptors [55], appears to be necessary for spinal dorsal horn sensitization (winding-up) and the behavioral hyperalgesia observed in pain syndromes. Notably, this relationship appears to be disrupted in σ1R^−/−^ mice in which the HINT1 protein is transferred to the NMDAR NR1 subunit and GPCRs are uncoupled from NMDARs. Thus, σ1R^−/−^ mice do not develop allodynia in response to partial sciatic nerve ligation, and they also show dampened NMDAR-mediated wind-up responses and ERK activation in the spinal cord [56]. Notably, CCI-induced allodynia and also morphine analgesia promotes the association of HINT1 proteins with NR1 subunits to the detriment of the MOR-NR1 interaction. Both situations augment NMDAR activity *via* GPCRs and thus, to prevent excitotoxicity the σ1R-HINT1 tandem regulates the extent of these associations. Accordingly, HINT1 protein switching to the NR1 subunits reduces the contribution of GPCRs to NMDAR-mediated allodynia and also, the negative control of MOR-recruited NMDARs over opioid analgesia. On morphine clearance, HINT1 again falls under the control of the MOR, although in neuropathies the transfer of HINT1 predominates until the influence of the activated GPCRs vanishes. This could be driven by GPCR antagonists or through the pharmacological regulation of the σ1R-HINT1 switch.

An interesting observation is that cerebral ischemia induces the accumulation of dopamine, serotonin and other neurotransmitters that contribute to neuronal death [57]. Accordingly, MOR antagonists, agents that deplete dopamine or serotonin at nerve terminals, and glutamate antagonists prevent or reduce the brain damage that results from experimental heatstroke [58, 59]. Notably, HINT1^−/−^ mice, in which GPCRs are uncoupled from NMDARs, exhibit much less glutamate NMDAR-mediated damage in response to ischemia than wild-type mice. Hence, it is possible that a series of focal-activated GPCRs help extend ischemic damage and that HINT1 is implicated in the positive influence that these GPCRs exert on NMDAR activity. Thus, the HINT1-σ1R tandem could regulate the interaction of NMDARs with a variety of GPCRs. In fact, the signaling proteins that sustain the functional interaction of the MORs and CB1 receptors with NMDARs also associate with other GPCRs, like HINT1, the RGSZ2-nNOS complex that regulates the interaction of HINT1 with NR1 subunits [28], and σ1Rs [44; and the present study].

Glutamate is the major excitatory neurotransmitter in the nervous system and deregulation of the NMDAR activity is associated with many neurological alterations, including neurodegenerative diseases [60], neuropathic pain [61, 62], mood disorders and psychosis/schizophrenia [63, 64]. However, it currently appears that GPCRs are implicated in such deregulation of NMDARs. Hence, the cross-regulation between GPCRs and NMDARs could account for the concurrent disturbances in NMDAR transmission and dopamine receptors associated with schizophrenia, or with serotonin receptors in major depression. The NMDAR-generated signals likely restrain those of dopamine receptors, such as D1 and D2, whereas NMDAR transmission contributes to the function of serotonin 5HT1A receptors. In subcortical areas the NMDAR negatively controls glutamatergic prefrontal cortical neurons. In this framework, a persistent NMDAR hypofunction could lead to secondary dopaminergic dysregulation like that seen in striatal and prefrontal brain regions of schizophrenic patients. The striatal NMDAR hypofunction would increase D2 receptor activity in this area, while in cortical neurons an enhanced glutamate release *via* presynaptic mGluRs diminishes dopamine discharge and, hence, D1 activity [65, 3]. In support of this hypothesis, experimental NMDAR hypofunction (induced by ketamine or PCP) causes glutamatergic hyperfunction and dopaminergic hypofunction in the prefrontal cortex. The higher hierarchy of NMDAR dysfunction in schizophrenia is suggested by the experimental antagonism of NMDA receptors, which induces psychotic symptoms and neurocognitive disturbances similar to schizophrenia [64, 65]. Likewise, NMDAR antagonism leads to rapid, robust, and relatively sustained antidepressant effects in patients with treatment-resistant major depression without the lag of onset of several weeks to months that is observed with traditional antidepressants [63, 66].

In the synapse, GPCRs could form complexes with NMDARs more frequently than actually suspected. Aminergic GPCRs must respond adequately to consecutive waves of their neurotransmitters, resensitizing within milliseconds; thus, their complexes with NMDARs would have a brief lifespan and, hence, be hardly detectable *ex vivo*. Coincidence in time or/and space determines the weight that the neural cell assigns to each of the incoming GPCR-mediated signals, and this weight is also influenced by the level of excitability that glutamate NMDARs confer to the postsynapse [12]. In this context, the interaction of GPCRs with NMDARs regulated by HINT1-σ1R deserves attention mostly because the main components of this switch have been described as vulnerability genes for schizophrenia [see 25, 67]. Moreover, HINT1^−/−^ mice show pro-psychotic behaviors and disturbed dopaminergic transmission [38, 39], and the σ1R has been implicated in neural alterations such as those observed in HINT1^−/−^ mice [32, 33, 34, 35].

The integrity of the HINT1-σ1R machinery is necessary to couple GPCR activity to that of the NMDARs but also, to disconnect their activities when necessary. We found that in association with CCI-induced allodynia and morphine-induced analgesia, the GPCR-NMDAR interaction was reduced to such a level that excitatory transmission did not compromise cell viability. Alternatively, when the cannabinoids that restrain NMDAR activity could provoke its hypofunction, the NMDAR must be disconnected from the influence of the GPCR and this disconnection is achieved by transfer of HINT1 from the GPCR to the NMDAR NR1 C1 subunit. The σ1R and its endogenous regulators, probably neurosteroids, play an essential role in this regulatory process, which serves to maintain NMDAR excitatory activity within physiological limits [28, 8].

The physiological relevance of this regulation is suggested by the release of pregnenolone in response to exogenous cannabinoids or opioids [68]. Most likely, following the conversion of pregnenolone into the σ1R antagonist progesterone, the NMDAR uncouples from the negative influence of cannabis-activated CB1 receptors, preventing hypoglutamatergia, which could lead to symptoms of psychosis. However, progesterone, by uncoupling MORs from NMDARs, prevents the recruitment of excessive NMDAR activity, diminishing the risk of excitotoxicity. Thus, excessive coupling of CB1 to NMDARs leads to glutamate hypofunction; this outcome could be caused by defects in the mechanism in charge of disconnecting both systems that comprise the HINT1-σ1R switch under regulation by calcium and ligands of the σ1R. In fact, the endocannabinoid system is another candidate for producing schizophrenia through NMDAR hypofunction [see 8, 67].

The pharmacology of pain control has already taken advantage of the HINT1-σ1R tandem even before its decisive role in the cross-regulation between GPCRs-NMDARs was defined. The σ1R was assigned a regulatory role for diverse proteins in the plasma membrane, NMDARs and MORs included [7, 69]. In this framework, the σ1R ligands that enhance morphine analgesia were classified as antagonists because they apparently lifted the σ1R-mediated negative control on MOR signaling. By contrast, those that increased morphine analgesia or that simply prevented the enhancing effect of the antagonists were classified as agonists [43]. A series of σ1R antagonists have been shown to potentiate MOR-mediated analgesia, to reduce the development of analgesic tolerance and more importantly, to reduce allodynia in animal models of neuropathic pain [43, 28, 70, 71, 72].

Our studies shed some light as to how σ1R regulates MOR-mediated analgesia and CCI-induced neuropathy [73, 46]. Thus, σ1R antagonists favor the transfer of HINT1 proteins to the NMDAR NR1 subunits and the disconnection of NMDARs from GPCRs, thereby reducing the impact of excitatory transmission on MOR function (enhancing opioid analgesia and dampening allodynia). Accordingly, in the presence of σ1R antagonists morphine analgesic tolerance develops more slowly. Moreover, σ1R antagonists can also rescue morphine analgesia from moderate tolerance [28, 72]. Some swapping of HINT1 proteins to the NMDARs occurs in the mice subjected to CCI, probably sustained by endogenous regulators of the HINT1-σ1R switch. Thus, it is possible that the exogenous antagonists of σ1R produce more efficient transfer of HINT1 proteins from activated GPCRs toward the NMDARs, thereby reducing the negative impact of allodynia on animal behavior.

The HINT1 protein displays nucleoside phosphoramidase and acyl-AMP hydrolase activity [74, 75]. The inhibition of HINT1 enzymatic activity enhances morphine analgesia and prevents the development of tolerance, and in mice suffering from CCI it alleviates mechanical allodynia. At the molecular level, HINT1 inhibition reduced the GPCR-mediated activation of NMDARs [76]. Thus, the σ1R and the HINT1 protein represent new promising therapeutic targets for pain pharmacology [76, 77] relevant to ongoing studies [78].

NMDAR antagonists usually fail trials in human beings because blocking synaptic NMDAR transmission hinders neuronal survival [79]. New approaches aimed at selectively targeting overactivated NMDARs are currently under development and clinical validation [2, 60]. Neuroactive steroids, such as pregnenolone, dehydroepiandrosterone and allopregnanolone, are altered in subjects with schizophrenia and bipolar disorder [80]. These adaptive alterations likely seek to regulate the HINT1-σ1R switch and rescue GPCR-mediated activation of NMDAR function. In this situation, regulators of σ1Rs and of HINT1 proteins [76, 28] could offer reliable and more efficacious treatments. Preliminary clinical trials with pregnenolone show the potential in to alleviate symptoms of schizophrenia [81], and synthetic σ1R antagonists have completed phase I safety and pharmacokinetic evaluations in humans [78]. Thus, the results from the present study indicate that palliative treatments for neuropathic pain and psychosis/schizophrenia directly targeting NMDAR of GPCR function could be complemented or even substituted with others directed to regulate the interrelation of GPCR-NMDAR.

## MATERIALS AND METHODS

### Animals, drugs and evaluation of antinociception

Male mice CD1 were used in this study. Knockdown mice: CD1 mice with targeted deletion of σ1R [28], and a mouse strain, 96% genetic background from 129 mice, with targeted disruption of HINT1 and wild-type littermate mice were also used. Experiments using animals were performed in accordance with the procedures for the Care and Use of Laboratory Animals of the European Commission guidelines (Directive 2010/63/EU). All the procedures for handling and sacrificing the animals were approved by the Committee on Animal Care at CSIC.

NMDA (#0114), MK801 (#0924), BD1047 (#0956), BD1063 (#0883), PRE084 (#0589), NE100 (#3133), SKF10047 (#1079) were all obtained from Tocris Bioscience (Bristol, U.K.). Morphine sulfate were obtained from Merck (Darmstadt, Germany), Progesterone (P7556) and Pregnenolone sulfate (P162) were purchased from Sigma-Aldrich (Madrid, Spain). Antinociception was expressed as a percentage of the maximum possible effect (MPE = 100 × [test latency-baseline latency] / [cut-off time (10 s) - baseline latency]). To facilitate a selective and straightforwardly access to their targets the compounds were each injected into the lateral ventricle of mice at 4 μL as previously described [82]. The response of the animals to nociceptive stimuli was assessed using the warm water (52°C) tail-flick test. The baseline latencies ranged from 1.7 to 2.0 seconds, and this parameter was not significantly affected by NMDA, the σ1R ligand (BD1047) or the solvent used: saline, 1.8 ± 0.2 seconds; and ethanol/Cremophor EL/physiological saline (1:1:18), 1.9 ± 0.2 seconds (*n* = 10). A cut-off time of 10 seconds was used to minimize the risk of tissue damage. Groups of 8 to 10 mice received a dose of morphine, alone or after the selected compounds. Analgesia was measured in the thermal tail-flick test at the peak effect for morphine analgesia, 30 min post-opioid treatment.

### Permanent focal cerebral ischemia and determination of infarct size

The middle cerebral artery (MCA) was exposed and occluded permanently by suture ligation [83]. A small craniotomy was made over the trunk of the right middle cerebral artery and above the rhinal fissure. The permanent MCA occlusion (MCAO) was done by ligature of the trunk just before its bifurcation between the frontal and parietal branches with 9-0 suture. Following surgery the mice were returned to their cages kept at room temperature and allowed free access to food and water. Complete interruption of blood flow was confirmed under an operating microscope.

For infarct size determination 48 hours after MCAO, magnetic resonance examination was performed using a BIOSPEC BMT 47/40 (Bruker, Ettlingen, Germany). Infarct volume was calculated using ImageJ 1.44l (NIH, Bethesda, MD, USA) from the T2-weighted images.

### Nerve injury pain model

After testing the basal mechanical sensitivity of the mice, neuropathic pain was induced by chronic constriction injury (CCI) under isoflurane/oxygen anesthesia [49]. Briefly, a 0.5 cm incision was made in the right mid-thigh, the biceps femoris muscle was separated and the sciatic nerve was exposed proximal to its trifurcation. Two ligatures were tied around the nerve, approximately 1 mm apart, until a short flick of the ipsilateral hind limb was observed. The incision was then closed with a 4-0 Ethicon silk suture in layers. The same procedure was used for sham surgeries except that the sciatic nerve was exposed but not ligated. The presence of allodynia (tactile pain threshold) was assessed using an automatic von Frey apparatus (Ugo Basile #37450, Comerio, Italy) on day 7 post-surgery as described [76].

### Bimolecular fluorescence complementation (BiFC) analysis

The pPD49.83 plasmid was used to generate two cloning vectors for BiFC analysis [24]. each construct containing: the heat shock promoter, hsp-16.41, a Myc or hemaglutinin tag to detect BiFC fusion proteins, a multiple cloning site to subclone the gene of interest, a linker sequence, and the N-terminal Venus fragment truncated at residue 173 (VN173) or the C-terminal Venus fragment from residue 155 (VC155) -a generous gift from Dr Chang-Deng Hu, Purdue University, USA. Full length murine NMDAR, σ1R, various GPCRs, HINT1, nNOS and RGSZ2 were all subcloned in frame into pCE-BiFC-VN173 or pCE-BiFC-VC155 plasmids using standard cloning strategies. The fidelity of the constructs was verified by sequencing. Chinese hamster ovary (CHO) cells were transfected (0.3 μg plasmids) using Lipofectamine 2000 (Invitrogen, Madrid, Spain) and incubated for 24 h prior to testing for transgenic expression. Samples were visualized on glass bottom plates (MatTek Co, Ashland, MA, USA) using a Leica DMIII 6000 CS confocal fluorescence microscope (Leica, Barcelona, Spain) equipped with a TCS SP5 scanning laser.

### Expression of recombinant proteins

The coding region of HINT1 (NM_008248), murine full-length (1-223) σ1R (AF004927), and the C-terminal regions of mus musculus MOR1 (AB047546: residues 286-398) and the glutamate receptor NMDAR1 (NM_008169) (residues 834-938), were all amplified by RT-PCR using total RNA isolated from mouse brains as the template. Specific primers containing an upstream Sgf I and a downstream Pme I restriction site were used, as described previously [27, 24, 28]. The PCR products were cloned downstream of the GST coding sequence and the TEV protease site. The sequenced proteins were identical to the GenBank™ sequences. The vector was introduced into E. coli BL21 (KRX #L3002, Promega, Madrid, Spain), and clones were selected on solid medium containing ampicillin. After overnight induction at room temperature (1 mM IPTG and 0.1% Rhamnose), the cells were collected by centrifugation, and the pellets were maintained at −80°C.

The purification of GST fusion proteins was done under native conditions on GStrap FF columns (GE#17-5130-01, Healthcare, Barcelona, Spain) and when necessary the fusion proteins retained were cleaved on the column with ProTEV protease (Promega, #V605A) and further purification was achieved by electroelution of the corresponding gel band (GE 200, Hoefer Scientific Instruments, San Francisco, CA, USA). The sequences were confirmed through automated capillary sequencing.

### *In vitro* interactions between recombinant proteins: pull-down of recombinant proteins, effect of calcium and neurosteroids and displacement assay

The association of HINT1 with either GST-tagged NR1 C-terminal sequence C0-C1-C2, MOR or σ1 receptor was studied. The HINT1 proteins were incubated either alone (negative control) or together with the GST tagged protein in 400 μL of a buffer containing 50 mM Tris-HCl, pH 7.4, 2.5 mM CaCl_2_ and 0.2% CHAPS and mixed by rotation for 30 min at RT. Subsequently, 40 μL glutathione Sepharose 4B (GE#17 0756 01; GE Healthcare, Barcelona, Spain) was added to the protein mixture, which was then recovered by centrifugation, washed three times, solubilized in 2x Laemmli buffer, and analyzed by Western blotting. The σ1R, NR1 C0-C1-C2, MOR or HINT1 proteins do not bind to GST (Z02039; GenScript Co., Piscataway, NJ).

The influence of added calcium on the association of σ1R with either NR1 C-terminal sequence C0-C1-C2 or MOR Ct sequence was also evaluated. The NR1 C-terminal sequence or MOR Ct was immobilized through covalent attachment to NHS-activated sepharose 4 fast flow (GE#17-0906-01) according to the manufacturer's instructions. The recombinant σ1R (100 nM) was incubated either alone (negative control) or together with the immobilized proteins in 200 μL of a buffer containing 50 mM Tris-HCl, pH 7.4 and 0.2% CHAPS in the presence of increasing amounts of calcium chloride for 30 min at RT. Parallel samples were used to evaluate the effect of neurosteroids (30 μM) on the σ1R/NR1 C0-C1-C2 or σ1R/MOR Ct association.

In a set of assays, the influence of σ1R on HINT1 association with either the NR1 C0-C1-C2 subunits or MOR Ct was determined through the preincubation of recombinant HINT1 (200 nM to obtain 100 nM of the functional dimer) with agarose-NR1 or agarose-MOR for 30 min with rotation at room temperature in 300 μL of 50 mM Tris-HCl, pH 7.5, 2.5 mM CaCl_2_, and 0.2% CHAPS. After the removal of the free HINT1, σ1Rs were added to the milieu. Agarose pellets containing the bound proteins were obtained by centrifugation, washed thrice, solubilized in 2x Laemmli buffer, and analyzed by Western blotting.

### Membrane preparations, immunoprecipitation and the detection of associated proteins

This procedure has been described elsewhere [84, 8]. Briefly, synaptosomal membranes were obtained from groups of 6 to 10 mice sacrificed by decapitation at various intervals after receiving icv injection of the compounds. The PAGs were collected and homogenized in 10 volumes of 25 mM Tris-HCl (pH 7.4), and 0.32 M sucrose supplemented with a phosphatase inhibitor mixture (P2850, Sigma-Aldrich, Madrid, Spain), H89 (B1427, Sigma-Aldrich) and a protease inhibitor cocktail (P8340, Sigma-Aldrich). The homogenate was centrifuged at 1000x*g* for 10 min to remove the nuclear fraction. The supernatant (S1) was centrifuged twice at 20,000x*g* for 20 min to obtain the crude synaptosomal pellet (P2). The final pellet was diluted in Tris buffer supplemented with a mixture of protease inhibitors (0.2 mM phenylmethylsulphonyl fluoride, 2 μg/mL leupeptin, and 0.5 μg/mL aprotinin), followed by division into aliquots and freezing at −80°C.

For immunoprecipitation studies, the PAG from 8 mice were typically pooled. The assays were repeated at least twice on samples receiving an identical treatment and collected at the same interval post-administration. To circumvent interference with signaling proteins attached to the cytosolic regions of the MOR, CB1R and NR1 subunits, antibodies were directed to their extracellular domains (GenScript Co., Piscataway, NJ, USA). The affinity purified IgGs against the extracellular domains of the MOR 2EL (205-216: MATTKYRQGSID; GenScript) [85, 86], CB1 EL (177-188: DFHVFHRKDSPN) [25] and the NMDAR NR1 subunit (483-496: KFGTQERVNNSNKK) [24] were labelled with biotin (Pierce #21217 & 21339, Thermo Scientific, Rockford, IL, USA). Negative controls were performed with IgGs heated for 10 min at 100°C or pre-absorbed with 0.1 mg of antigenic peptide for 1 h at room temperature. Pilot assays were performed to adjust the amount of IgGs and sample protein, and to determine the incubation period required to precipitate the desired protein in a single run. Thus, in any second precipitation only residual target signal would be evident. The target proteins were subsequently immunoprecipitated from solubilized membranes and resolved by SDS/polyacrylamide gel electrophoresis (PAGE), as described previously. To confirm the selectivity of the co-immunoprecipitated proteins, thereby ruling out any possibility of interactions occurring during the solubilization/immunoprecipitation procedure, brain synaptosomal membranes were heated at 100° C for 10 min in 40 mM Tris-HCl, 1% SDS buffer. This mixture was then cooled to room temperature and to allow the IgGs to bind to their target proteins, the SDS concentration was reduced by adding octylthioglucoside to a final percentage of 0.65%. Under these conditions, the target protein did not associate with those observed in the co-immunoprecipitations assays. The complete procedure has already been described elsewhere [84, 87].

The separated proteins were subsequently transferred onto 0.2 μm polyvinylidene difluoride (PVDF) membranes (#162-0176, BioRad, Madrid, Spain) and probed overnight at 6°C with the primary antibodies diluted in Tris buffered saline (pH 7.7) + 0.05% Tween-20, followed by detection with secondary antibodies (2 h) conjugated to horseradish peroxidase. Antibody binding was visualised through chemiluminescence (#170-5061, BioRad, Madrid, Spain) and recorded with a ChemiImager IS-5500 (Alpha Innotech, San Leandro, California). Densitometry was performed using the Quantity One Software (BioRad) and expressed as the mean of the integrated volume (average optical density of the pixels within the object area/mm^2^). Co-precipitation studies: equal loading among related samples was verified after determining the target protein in parallel blots of the same immunoprecipitated samples. The primary antibodies included anti-σ1R (#42-3300, Invitrogen, Barcelona, Spain) [67]; anti-MOR CT [86, 88]; anti-NMDAR1 (#MAB1586, Merk-Millipore, Madrid, Spain) [27]; anti-NMDAR1 C1 (#MAB5046P; Merck-Millipore) [25]; anti-NMDAR1 C2 (#MAB5048P; Merck-Millipore) [27]; anti-NMDAR2A (#ab14596; Abcam, Cambridge, UK) [27]; antiNMDAR2B (#ab14400; Abcam) [27] and antiNMDAR3B (#ab2639; Abcam) anti-PKCI/HINT1 (#H00003094-A01, Abnova, TaipeiCity, Taiwan) [27].

### Artwork and statistical analysis

Graphs and Statistical analysis of the data was carried out using the Sigmaplot/SigmaStat v.13 package (SPSS Science Software, Erkrath, Germany). Significance was defined as *p* < 0.05.
